# Association between ADAM12 Single-Nucleotide Polymorphisms and Knee Osteoarthritis: A Meta-Analysis

**DOI:** 10.1155/2017/5398181

**Published:** 2017-08-08

**Authors:** Zheng-tao Lv, Shuang Liang, Xiao-jian Huang, Peng Cheng, Wen-tao Zhu, An-min Chen

**Affiliations:** Department of Orthopedics, Tongji Hospital, Tongji Medical College, Huazhong University of Science and Technology, Wuhan, Hubei 430030, China

## Abstract

**Objective:**

ADAM12 polymorphisms may be associated with the risk of knee osteoarthritis (KOA), but currently available evidence remains controversial. We performed this meta-analysis to confirm whether ADAM12 polymorphisms were associated with susceptibility of KOA.

**Methods:**

A comprehensive literature search in PubMed, EMBASE, and ISI Web of Science was conducted to identify observational studies assessing the association between ADAM12 polymorphisms and susceptibility of KOA. The strength of association was indicated as odds ratio (OR) and the corresponding 95% confidence interval (95%CI). Four types of genetic model (additive model, dominant model, recessive model, and allele model) were evaluated for each included study. Subgroup analysis by ethnicity was performed.

**Results:**

Seven case-control studies comprising a total of 3512 KOA patients and 5405 healthy controls were included in the meta-analysis. A significant association between rs1871054 and increased KOA risk was found in each genetic model. No significant association was found between KOA and rs3740199, rs1044122, or rs1278279 in any genetic model.

**Conclusion:**

Based on the findings of our study, there was a modest but statistically significant association between rs1871054 and risk of KOA in Asian population, while other polymorphisms (rs3740199, rs1044122, or rs1278279) in ADAM12 were not associated with KOA in any population.

## 1. Introduction

Osteoarthritis (OA) is the most common chronic and progressive musculoskeletal disease of the elderly all around the world and has been identified in all ages. It is characterized by synovitis, thickening of the joint capsule, progressive degradation of articular cartilage, joint space narrowing, osteophyte formation, and subchondral sclerosis, resulting in chronic joint pain and loss of function of the elderly individuals [1, 2]. The risk factors of OA are multifactorial, like age, sex, body weight, hormonal status, trauma, family history, selected activation, and so forth [1, 3]. In addition to the risk factors above, numerous genome-wide association studies have suggested that a mass of genes contribute to the occurrence of OA in recent years [4].

ADAM (a disintegrin and metalloprotease), a kind of type-I transmembrane and soluble glycoproteins, is involved in the process of signal transduction, cell adhesion, migration, and proteolysis [5–7]. As one of the members of ADAM family, ADAM12 is associated with the pathological processes of a variety of cancers [8–17], liver fibrosis [18], asthma [19], and hypertension and cardiac hypertrophy [20]. Several studies have demonstrated that ADAM12 played a pivotal role in chondrocyte proliferation and maturation, osteoclast formation, and bone formation [21, 22]. Additionally, the serum levels of ADAM12-S protein have high relationship with the degree of knee osteoarthritis (KOA) [23], indicating the potential relationship of them.

The single-nucleotide polymorphisms (SNPs) within the coding region can alter the amino acid sequence of a protein and influence the function of the corresponding protein. The association of ADAM12 SNPs with the susceptibility to the KOA could provide a new direction in KOA research. Although several studies have suggested that ADAM12 SNPs can influence the susceptibility to knee osteoarthritis but no consensus have been achieved. To our knowledge, no meta-analysis has been carried out to assess the correlation between ADAM12 polymorphisms and susceptibility to KOA. Therefore, we conducted this meta-analysis aiming to assess the association between the ADAM12 SNPs and KOA.

## 2. Methods

This meta-analysis was performed complying with the PRISMA guideline [24].

### 2.1. Literature Search

Eligible studies were retrieved by searching PubMed, EMBASE, and ISI Web of Science. All the online databases were searched up to October 2016 without language restriction. A combination of Medical Subject Heading (MeSH) and free text words was used: (osteoarthritis or osteoarthrosis or osteoarthritides or “Osteoarthritis” [Mesh]) and (“ADAM12 Protein” [Mesh] or ADAM12 or meltrin-alpha) and (“Polymorphism, Single Nucleotide” [Mesh] or Single Nucleotide Polymorphism or SNP or SNPs). In addition, we screened the reference lists of relevant reviews to avoid missing eligible studies.

### 2.2. Inclusion Criteria

Data were collected from published articles, and conference and meeting abstracts were consequently excluded. The studies were included only if they met the following criteria: (1) case-control or cohort studies investigating the association between ADAM12 polymorphism and KOA; (2) KOA should be diagnosed according to the American College of Rheumatology criteria, imaging records or total knee replacement due to primary KOA; (3) healthy controls were required to have no symptoms or signs of KOA, and the genotype distribution of control population should meet the Hardy-Weinberg equilibrium (HWE); (4) included studies should provide odds ratio (OR) and the associated 95% confidence interval (95% CI) or detailed information about genotype frequency. If studies with overlapping population were identified, the most complete one was included in our meta-analysis.

### 2.3. Data Extraction

Two investigators (Z. Lv and S. Liang) screened each article independently and were blinded to the results of the other reviewer. Two reviewers performed a stringent screening to determine eligible studies independently based on the predesigned inclusion criteria. Data were extracted independently by two reviewers from these selected articles using a standardized collection form, which included first author, country, year of the publication, study design, ethnicity, sample sizes, genotyping in case and control groups, and HWE of control groups. Any disagreement between the two reviewers was resolved through discussion until a consensus was reached. The third review author (A. Chen) was consulted if a consensus could not be reached.

### 2.4. Quality Assessment

The Newcastle-Ottawa Scale (NOS) [25] for the assessment of nonrandomized studies was used to assess the methodological qualities of case-control and cohort studies. Two reviewers assessed the methodological qualities of included studies independently, and the results of risk of bias judgement were compared afterwards.

### 2.5. Statistical Analysis

For the observed genotype frequencies in control group of each included study, Chi^2^ test was applied for the HWE. The OR of ADAM12 polymorphisms (rs3740199, rs1871054, rs1044122, and rs1278279) and OA risk was evaluated for each study, and the strength of association was indicated as OR and the corresponding 95% CI. The additive model was selected as the primary genetic model because it is a robust screen of a variety of other genetic models. Additionally, we determined the association for other possible genetic models: dominant model, recessive model, and allele model. Heterogeneity among studies was estimated using a *Q*-test and the Higgins *I*^2^ test (*P* > 0.1 and *I*^2^ < 50% indicate acceptable heterogeneity). We combined the OR of each study using fixed-effect model if there was no evident between-study heterogeneity across the eligible comparisons. Otherwise, a random-effect model was employed.

To evaluate the ethnicity-specific effect on the association between ADAM12 polymorphisms and OA risk, subgroup analysis by ethnicity (Asian/Caucasian) was subsequently performed. Begg's rank correlation test and Egger's linear regression test using Stata version 12.0 (Stata Corp LP, USA) were used to assess the publication bias if the number of included studies were larger than five. Publication bias was considered present with *P* < 0.05.

## 3. Results

### 3.1. Literature Search Results

**Figure 1:**
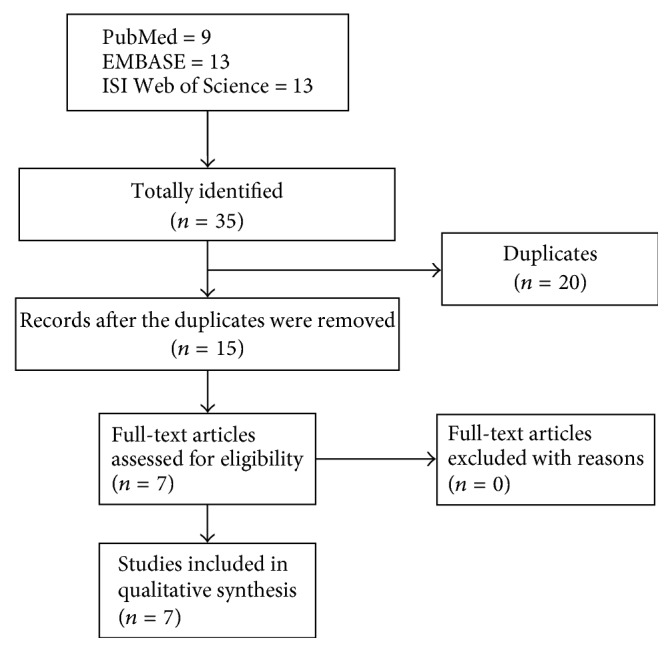
Flow chart of literature search.

The literature selection process was presented in Figure 1. The preliminary search yielded 35 potentially eligible records including 9 from PubMed, 13 from EMBASE, and 13 from ISI Web of Science. 20 records were removed because they were duplicated for retrieval, and the remaining 15 studies were screened with titles and abstracts. Three studies were removed because they were conference abstracts, and five records were deleted because they were not relevant to ADAM12 or OA. Finally, seven studies [3, 26–31] entered the full-text screen stage, all of which were included in our meta-analysis.

### 3.2. Main Characteristics of Included Studies

Seven case-control studies [3, 26–31] involving a total of 3512 KOA patients and 5405 healthy controls were included in our study. There were four studies [3, 27, 29, 31] on Asian population and three studies [26, 28, 30] in Caucasian population. As expected, the genotypes distribution of SNP rs3740199, rs1871054, rs1044122, and rs1278279 was in agreement of HWE in all included studies. The main characteristics and genotypes distribution of ADAM12 polymorphisms were listed in Table 1.

### 3.3. Quality Assessment

The NOS based on eight items was employed to assess the methodological quality of included studies. The OA cases were diagnosed according to ACR criteria or radiographical examination, and patients that have underwent total knee replacement were also included. However, representativeness of cases in our selected studies was not sufficient because only one study [27] recruited consecutive patients. The definition of controls was reported in detail by all studies. Regarding the comparability of cases and controls on the basis of the design or analysis, three studies [27, 30, 31] enrolled age-matched healthy controls. The detailed information about quality assessment was listed in Table 2.

### 3.4. Meta-Analysis Results

**Figure 2:**
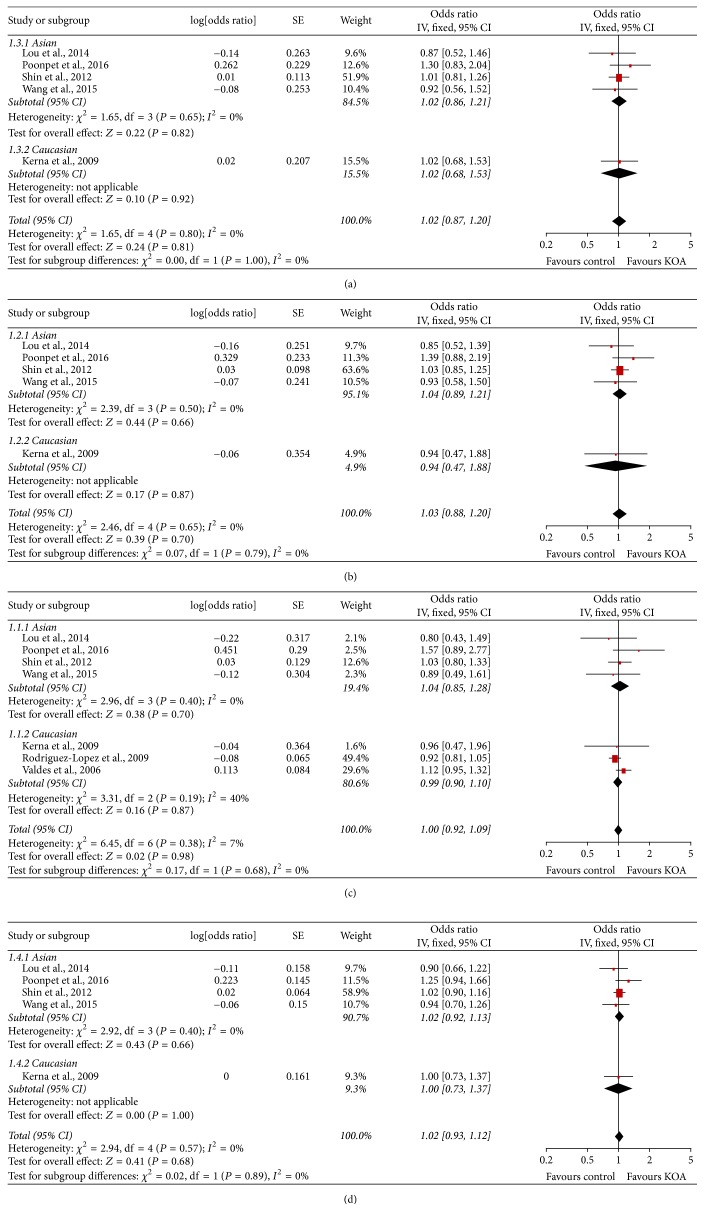
Forest plots of rs3740199 and knee osteoarthritis, subgroup by ethnicity: (a) additive model; (b) dominant model; (c) recessive model; and (d) allele model.

**Figure 3:**
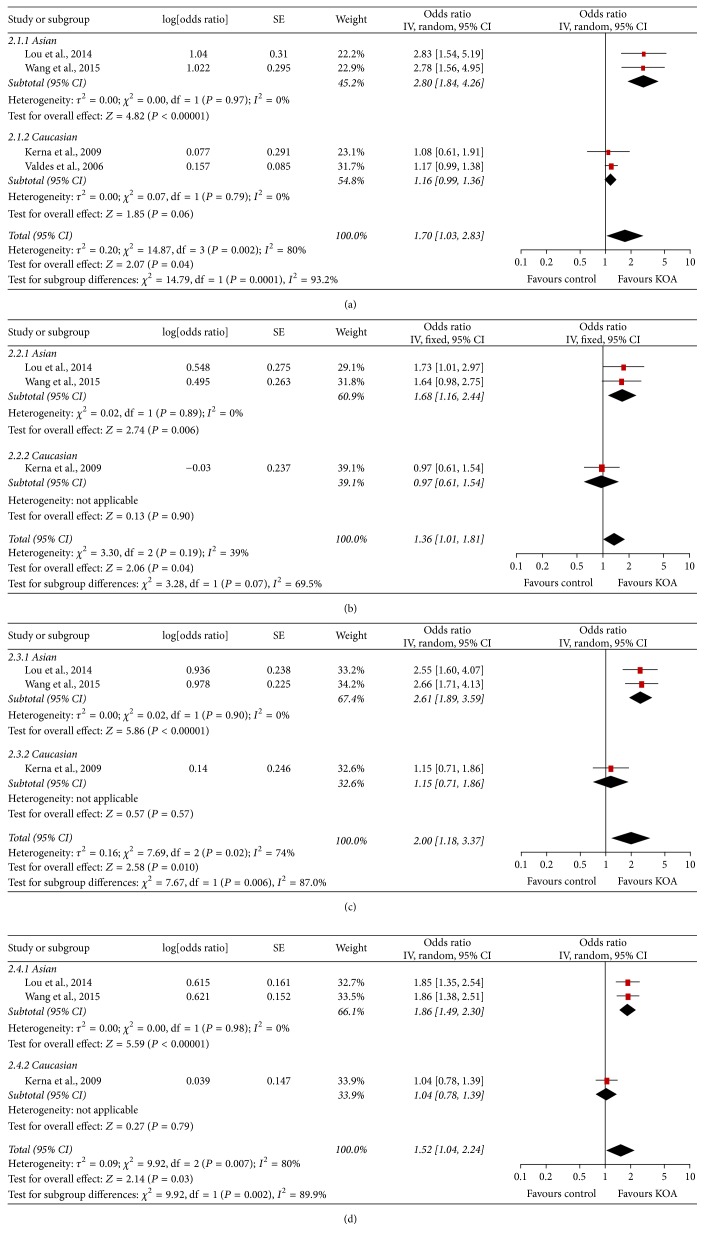
Forest plots of rs1871054 and knee osteoarthritis, subgroup by ethnicity: (a) additive model; (b) dominant model; (c) recessive model; and (d) allele model.

**Figure 4:**
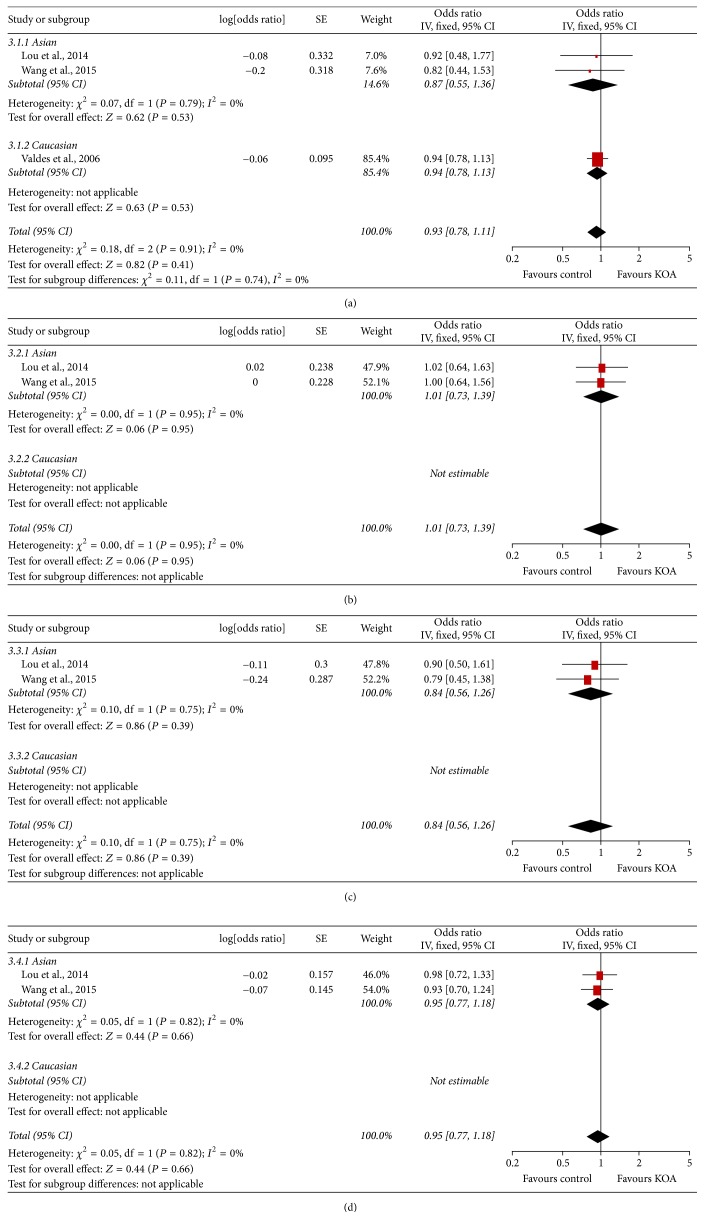
Forest plots of rs1044122 and knee osteoarthritis, subgroup by ethnicity: (a) additive model; (b) dominant model; (c) recessive model; and (d) allele model.

**Figure 5:**
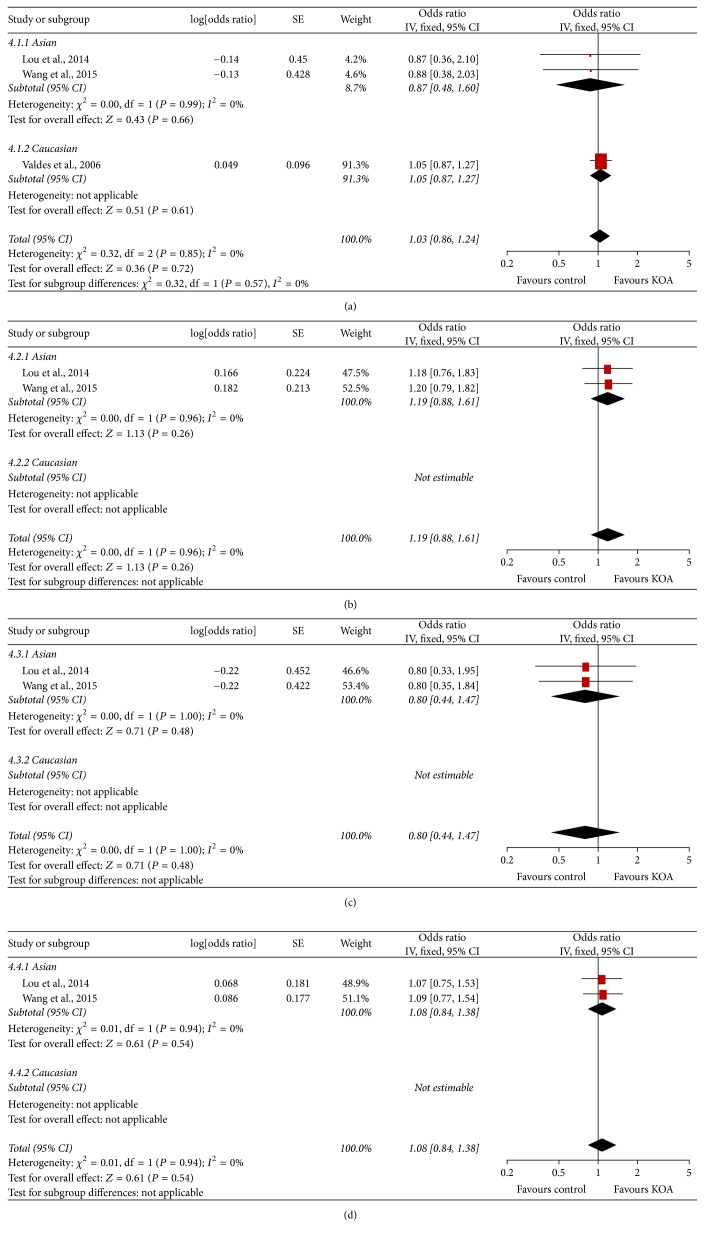
Forest plots of rs1278279 and knee osteoarthritis, subgroup by ethnicity: (a) additive model; (b) dominant model; (c) recessive model; and (d) allele model.

The meta-analysis findings of the association between ADAM12 SNPs and KOA risk were presented in Figures 2, 3, 4 and 5. The pooled ORs were calculated for additive model contrast, dominant model contrast, recessive model contrast, and allelic contrast. The ADAM12 SNP rs1871054 was found to be significantly associated with increased KOA risk in additive model (OR 1.70, 95% CI 1.03, 2.83; *I*^2^ = 80%), dominant model (OR 1.36, 95% CI 1.01, 1.81; *I*^2^ = 39%), recessive model (OR 2.00 95%, CI 1.18, 3.37; *I*^2^ = 74%), and allele model (OR 1.52, 95% CI 1.04, 2.24; *I*^2^ = 80%). The polymorphisms rs3740199, rs1044122, and rs1278279 were found with no statistical association with KOA risk no matter for which genetic model.

### 3.5. Subgroup Analysis and Publication Bias

Subgroup analysis by ethnicity was undertaken to determine whether there was a population-dependent effect on the association between ADAM12 SNP and KOA risk. There was no significant association between polymorphisms rs3740199, rs1044122, rs1278279, and OA risk in all populations. In subgroup analysis based on ethnicity, no statistically significant association was found between rs3740199, rs1044122, rs1278279, and OA risk in either Caucasian or Asian populations. With regard to rs1871054, subgroup analysis by ethnicity revealed that rs1871054 was significantly associated with OA risk among Asian populations: additive model (OR 2.80, 95% CI 1.84, 4.26; *I*^2^ = 0%), dominant model (OR 1.68, 95% CI 1.16, 2.44; *I*^2^ = 0%), recessive model (OR 2.61, 95% CI 1.89, 3.59; *I*^2^ = 0%), and allele model (OR 1.85, 95% CI 1.49, 2.30; *I*^2^ = 0%), but not among Caucasian populations.

Publication bias was only assessed in polymorphism rs3740199 because the number of included studies was greater than five. Begg's test (additive model: *z* = 0.00, *P* = 1.00; dominant model: *z* = 0.73, *P* = 0.46; recessive model: *z* = 0.73, *P* = 0.46; allele model: *z* = 0.73, *P* = 0.46) and Egger's test (additive model: *t* = 0.89, *P* = 0.42; dominant model: *t* = −0.03, *P* = 0.98; recessive model: *t* = 0.06, *P* = 0.96; allele model: *t* = 0.00, *P* = 1.00) showed no significant publication bias.

## 4. Discussion

In this meta-analysis we collected seven case-control studies with a total of 3512 KOA patients and 5405 healthy controls, and no restriction on ethnicity and language was imposed. Meanwhile, we performed subgroup analyses to identify population-dependent effect on the association between ADAM12 SNPs and KOA risk. The analysis results of this study indicated a modest but statistically significant association between rs1871054 in ADAM12 and KOA in Asian population but not in Caucasian population. In addition, no significant correlation was found between risk of KOA and rs3740199, rs1044122, or rs1278279 in any population. No evidence was found for publication bias in the meta-analysis for rs1871054 in any genetic model. But, due to the limited number of included studies, funnel plots were not generated for meta-analyses of rs3740199, rs1044122, and rs1278279.

ADAM12, located on chromosome 10q26.2, is a member of the Zn^2+^ dependent metalloproteinase superfamily that have been implicated in a variety of biological processes involving cell-cell and cell-matrix interactions [32, 33], cell adhesion and fusion [33–35], and extracellular matrix restructuring [33]. It was demonstrated that ADAM12 plays an important role in introducing the formation of giant cell and osteoclast, and this may affect the progress of bone remodeling in KOA development. And the addition of antisense ADAM12 messenger RNA caused a 70% inhibition of osteoclast-like cells [36], which may explain the correlation between this gene and the osteophyte formation. It was reported that the expression of ADAM12 may be involved in the cleavage of insulin-like growth factor binding protein 5 (IGFBP-5), which decreases bioavailability of insulin-like growth factor-1 (IGF-1), resulting in the inhibition of chondrocyte proliferation [37]. Therefore, the change of ADAM12 SNPs may contribute to the loss of articular cartilage. Moreover, ADAM12 has been shown to be important in mediating growth factor shedding and cell adhesion and fusion [33, 35], which also indicates its role in the synovitis of KOA. Meanwhile, animal studies suggested that the overexpression of ADAM12 could promote the inflammatory response and accelerate synovial fibrosis [38]. These evidences all indicated that the polymorphisms of ADAM12 may be important in the progress of KOA.

ADAM12 gene contains multiple SNPs including rs1871054, rs3740199, rs1044122, and rs1278279. As indicated above, only SNP rs1871054 was confirmed to be associated with a modestly increased risk of KOA in Asian but not Caucasian population. The pooled ORs for additive model contrast, dominant model contrast, recessive model contrast, and allelic contrast all suggested that rs1871054 was associated with an increased risk for KOA. When we restricted the ethnicity to Asian ethnicity, the between-study heterogeneity was low in any genetic model, suggesting that ethnicity might be one of the potential sources of heterogeneity among studies. And the difference of genetic effects between Caucasian and Asian populations may be the outcomes of gene-environment or gene-gene interactions, suggesting that the genetic effect of rs1871054 polymorphisms is stronger in Asia than in Caucasian population. Additionally, different criteria were used to define respective cases, which could also increase the heterogeneity in different ethnic populations.

Several potential limitations in our meta-analysis should not be ignored. Firstly, only seven case-control studies were included in this meta-analysis. The sample size was not large enough to exactly validate the correlation between the ADAM12 polymorphisms and susceptibility to KOA. Therefore, more studies with larger sample sizes are required, so as to guarantee the stability and reliability of the meta-analysis. Secondly, the association between SNPs in ADAM12 and susceptibility to KOA may be affected by other confounding factors, such as age, gender, and BMI. We are unable to perform further stratified analysis due to the incomplete raw data. For instance, KOA is known to be more prevalent in females than in males, and future studies should provide detailed allele frequencies and genotypes in both genders. Thirdly, KOA is associated with both genic and environmental risk factors, including body weight, hormonal status, trauma, family history, and selected activation. But the studies included in this meta-analysis did not control for these mixed factors.

In conclusion, our meta-analysis suggested that ADAM12 rs1871054 polymorphism is associated with the susceptibility to KOA in Asian but not Caucasian population. There was insufficient evidence to support an association between rs3740199, rs1044122, rs1278279, and KOA. Further case-control studies with large sample size should be performed to confirm our conclusion. Despite the existence of limitation, this study provided an important evidence for the identification of the association between ADAM12 SNPs and KOA.

## 5. Conclusion

In summary, the results of our current study showed that the polymorphism rs1871054 within ADAM12 was significantly associated with increased risk of KOA and the association may be population dependent, currently only observed in Asian population. In either Asian population or Caucasian population, no statistically significant association between rs3740199, rs1044122, rs1278279, and KOA risk was found. Large-scale studies in different populations are encouraged.

## Figures and Tables

**Table 1:** Allele and genotypes distribution of ADAM12 polymorphisms in cases and controls.

Study	Country	Ethnicity	Study design	Case-control	Case			Control			HWE
rs3740199					CC	CG	GG	CC	CG	GG	
Kerna et al., 2009	Estonia	Caucasian	Case-control	66/123	81	66	16	106	89	20	0.37
Lou et al., 2014	China	Asian	Case-control	152/179	32	78	42	42	93	44	0.60
Poonpet et al., 2016	Thailand	Asian	Case-control	200/200	56	102	42	46	100	54	0.98
Rodriguez-Lopez et al., 2009	Spain	Caucasian	Case-control	1602/2370	NA	NA	NA	NA	NA	NA	>0.05
Shin et al., 2012	Korea	Asian	Case-control	725/1737	147	364	214	350	863	524	0.88
Valdes et al., 2006	UK	Caucasian	Case-control	603/596	NA	NA	NA	NA	NA	NA	>0.10
Wang et al., 2015	China	Asian	Case-control	164/200	36	84	44	47	102	51	0.77
rs1871054					CC	TC	TT	CC	TC	TT	
Kerna etal., 2009	Estonia	Caucasian	Case-control	66/123	42	79	42	50	111	54	0.63
Lou et al., 2014	China	Asian	Case-control	152/179	69	57	26	44	88	47	0.83
Valdes et al., 2006	UK	Caucasian	Case-control	603/596	NA	NA	NA	NA	NA	NA	>0.10
Wang et al., 2015	China	Asian	Case-control	164/200	76	59	29	49	99	52	0.89
rs1044122					CC	TC	TT	CC	TC	TT	
Lou et al., 2014	China	Asian	Case-control	152/179	24	81	47	31	92	56	0.52
Valdes et al., 2006	UK	Caucasian	Case-control	603/596	NA	NA	NA	NA	NA	NA	>0.10
Wang et al., 2015	China	Asian	Case-control	164/200	25	88	51	37	101	62	0.71
rs1278279					AA	AG	GG	AA	AG	GG	
Lou et al., 2014	China	Asian	Case-control	152/179	9	59	84	13	60	106	0.32
Valdes et al., 2006	UK	Caucasian	Case-control	603/596	NA	NA	NA	NA	NA	NA	>0.10
Wang et al., 2015	China	Asian	Case-control	164/200	10	62	92	15	64	121	0.12

NA: not available; HWE: Hardy-Weinberg equilibrium.

**Table 2:** Quality assessment of included studies.

Item/study	Kerna et al., 2009	Lou et al., 2014	Poonpet et al., 2016	Rodriguez-Lopez et al., 2009	Shin et al., 2012	Valdes et al., 2006	Wang et al., 2015
Adequate definition of cases	*∗*	*∗*	*∗*	*∗*	*∗*	*∗*	*∗*
Representativeness of cases	—	*∗*	—	—	—	—	—
Selection of control subjects	—	—	—	—	—	—	—
Definition of control subjects	*∗*	*∗*	*∗*	*∗*	*∗*	*∗*	*∗*

Control for important factor or additional factor	—	*∗*	—	—	—	*∗*	*∗*

Exposure assessment	*∗*	*∗*	*∗*	*∗*	*∗*	*∗*	*∗*
Same method of ascertainment for all subjects	*∗*	*∗*	*∗*	*∗*	*∗*	*∗*	*∗*
Nonresponse rate	*∗*	*∗*	*∗*	*∗*	*∗*	*∗*	*∗*

A study could be awarded a maximum of one *∗* for each item except for the item “control for important factor or additional factor.” The definition/explanation of each column of the Newcastle-Ottawa Scale is available from http://www.ohri.ca/programs/clinical_epidemiology/oxford.asp.
